# Sources of Cancer Neoantigens beyond Single-Nucleotide Variants

**DOI:** 10.3390/ijms231710131

**Published:** 2022-09-04

**Authors:** Aude-Hélène Capietto, Reyhane Hoshyar, Lélia Delamarre

**Affiliations:** Cancer Immunology Department, Genentech, South San Francisco, CA 94080, USA

**Keywords:** alternative source of neoantigen, cancer, gene fusion, RNA splicing, frameshift, dark matter

## Abstract

The success of checkpoint blockade therapy against cancer has unequivocally shown that cancer cells can be effectively recognized by the immune system and eliminated. However, the identity of the cancer antigens that elicit protective immunity remains to be fully explored. Over the last decade, most of the focus has been on somatic mutations derived from non-synonymous single-nucleotide variants (SNVs) and small insertion/deletion mutations (indels) that accumulate during cancer progression. Mutated peptides can be presented on MHC molecules and give rise to novel antigens or neoantigens, which have been shown to induce potent anti-tumor immune responses. A limitation with SNV-neoantigens is that they are patient-specific and their accurate prediction is critical for the development of effective immunotherapies. In addition, cancer types with low mutation burden may not display sufficient high-quality [SNV/small indels] neoantigens to alone stimulate effective T cell responses. Accumulating evidence suggests the existence of alternative sources of cancer neoantigens, such as gene fusions, alternative splicing variants, post-translational modifications, and transposable elements, which may be attractive novel targets for immunotherapy. In this review, we describe the recent technological advances in the identification of these novel sources of neoantigens, the experimental evidence for their presentation on MHC molecules and their immunogenicity, as well as the current clinical development stage of immunotherapy targeting these neoantigens.

## 1. Introduction

The clinical success of immune checkpoint blockade (ICB) therapy has revealed that T cells are the primary mediators of anti-tumor immunity. The tumor antigens that drive protective T cell responses have been elusive until about 10 years ago when it was discovered that somatic mutations that accumulate in cancers can stimulate both CD8 and CD4 T cell responses [1,2] and are associated with clinical response to ICB therapy [3].

Somatic mutations can translate into mutated proteins, and thus generate novel antigens or neoantigens that are recognized as foreign/non-self by the immune system and elicit potent T cell responses against cancer cells. Peptides derived from these proteins can enter the major histocompatibility complex (MHC) pathways for presentation to T cells. Endogenous proteins are processed by the proteasome into peptides that enter the endoplasmic reticulum and are loaded onto MHC class I (MHCI) molecules before being transported to the cell surface for presentation to CD8 T cells. Alternatively, peptides can also be presented on MHCII to CD4 T cells. They are generally derived from exogenous proteins and are generated by lysosomal proteases and loaded on MHCII in the endosomal/lysosomal compartments [4].

Only a minority of peptide-derived proteins are presented on MHC, and antigen prediction is critical to the development of successful antigen-targeted immunotherapies. The earliest antigen prediction tools relied on peptide binding prediction on MHC molecules, and provided enrichment for immunogenic peptides [5]. However, these prediction methods also had high false positive rates [6]. More recently, improved mass spectrometry technologies associated with comprehensive genomic analyses facilitated the generation of large datasets of naturally processed antigens presented on a broad range of MHC alleles, also called human leukocyte antigen (HLA) in humans [7,8,9]. These new datasets allowed for incorporation of peptide processing and presentation and led to the significant improvement of prediction algorithms [10,11,12,13,14,15,16]. In addition, the generation of immunopeptidomics data from monoallelic cell lines allowed clear identification of eluted peptides for specific HLA types compared to multiallelic datasets that require deconvolution [17]. This strategy boosted the detection of peptide/HLA-I complexes for low-frequency HLA-Is, improving the patient’s coverage of neoantigen-prediction HLA-I presentation tools [18]. For HLA-II molecules, however, progress has been slower, mostly due to the higher polymorphism of HLA-II molecules, the more variable length of the bound peptides, and the complexity of peptide/HLA-II interaction. The field is currently amplifying the efforts to improve the limited accuracy of HLA-II neoantigen prediction algorithms, notably by increasing training data from mass spectrometry [19,20].

**Figure 1:**
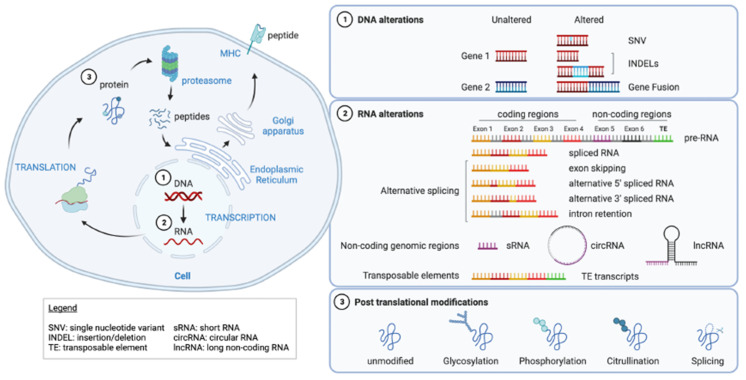
Sources of neoantigens (created with Biorender.com (accessed on 22 August 2022)).

Single-nucleotide variants (SNV) and small insertion/deletion mutations (indels) are currently favored targets for neoantigen-specific immunotherapy approaches. However, the tumor mutational burden may not be sufficient to mount an efficacious anti-tumor response in many cancers. In addition, the number of SNV-predicted neoantigens reported to be immunogenic is low (~3%), as shown in the meta-analysis of 13 different publications [21]. While the newest advances in computational predictions may reflect a better ratio, it is clear that key determinants of immunogenicity are not fully understood and/or yet incorporated in neoantigen prediction models. As TCR recognition is critical to T cell activation, more disruptive aberrations than [SNVs/small indels] may generate qualitatively distinct T cell responses. Cancer cells accumulate various other types of alterations at the DNA, RNA, and protein levels that are not observed in normal cells, and may be an alternative source of cancer neoantigens with a more favorable profile (Figure 1). At the DNA level, gene fusions have been largely ignored as a source of neoantigens. At the RNA level, variants include alternative splicing events (ASE) and dysregulation of transposable element (TE) expression. Post-translational modifications include spliced peptides, glycopeptides, phosphopeptides, or citrullinated peptides.

In this review, we describe the recent updates on identification and prediction methods, as well as preclinical and clinical studies showing promising results for most of the DNA/RNA aberrations as novel sources of neoantigens.

## 2. DNA Alterations

### 2.1. SNV/Indels

SNVs and small indels are abundant in many cancers [22] and are used as the reference in neoantigen-targeted therapy. 

Identification of SNV and small indels through NGS techniques and softwares is highly accurate [23], and a plethora of [SNV/small indel]-derived neoantigen predicting tools have been published in the last decade [23,24,25] with significant increased performance. However, the immunogenic fraction of predicted neoantigen candidates remains small even with improved presentation predictions, suggesting that other critical determinants are required for immunogenicity (Table 1 and Table 2).

Recent advances have focused on further identifying critical physicochemical and structural properties of immunogenic mutated peptides that led to TCR binding and T cell activation. Studies found that hydrophobicity, amino acid charge, and size of the MHCI peptide impacts TCR binding. In addition, it is important to consider the recognition of the presented peptide as foreign by the immune system. Features such as divergence between mutated (MUT) and wild-type (WT) epitopes, and similarity to microbial antigens have been suggested to contribute to peptide immunogenicity [26,27,28,29,30,31,32,33,34]. Our work showed that mutations in direct contact with the TCR were more likely to be immunogenic [32,35]. In addition, mutations at anchor positions that increase binding affinity to MHCI in comparison to the corresponding WT peptide may potentially result in novel and more immunogenic epitopes. Indeed, the difference in binding affinity between the MUT peptide and the corresponding WT (relative binding affinity) is an important predictor of immunogenicity [35,36]. Further development of artificial intelligence methods will certainly help integrate all these features into one neoantigen prediction pipeline workflow, and likely further increase immunogenicity prediction accuracy [36]. However, it is important to note that SNVs differ from the WT counterpart by only one amino acid, which may not always be sufficient to differentiate from the self and elicit potent immune responses. Indeed, we found a high frequency of T cell cross-reactivity between MUT peptides and their WT counterpart [35]. More disruptive aberrations may have a higher potential to generate a qualitative T cell response. While superior quality per se of the T cell response for non-SNV neoantigens remains to be proven, frameshift indels that generate long novel AA sequences can lead to a higher number of HLA epitopes presented on a more diverse set of HLA alleles than SNVs [37,38,39]. In addition to a higher rate of neo-epitope candidates per mutation, [long] indels-based immunotherapy may also be less subject to HLA loss-induced cancer immune evasion. 

### 2.2. Gene Fusion

Chromosomal translocations or deletions induce DNA rearrangement that can lead to the fusion of genes, thus possibly generating fusion proteins (Figure 1). Some of these newly formed fusion products have been identified as oncogenes, such as BCR-ABL in leukemias [40,41], and used as diagnostic or predictive biomarkers as well as drug targets [42]. These oncogenic fusion proteins are also attractive neoantigen targets due to high dissimilarity to self, clonality, as well as being shared between patients (Table 1).

While more than 260 gene fusions have been reported in hematological disorders, only 70 have been identified in solid tumors [43]. Karyotypes from solid tumors are more complex, resulting in poor quality and/or less accurate cytogenetic analysis to identify chromosomal abnormalities [43]. With the development of Fluorescence In Situ Hybridization (FISH), fusion breakpoints could be detected at the molecular level and this led to new structural rearrangement discoveries. However, FISH assay is not suitable for high-throughput screening due to live material requirement, cost, and probe specificity. It is only with the advances of targeted sequencing techniques that increased reliable identification of gene fusion events could be achieved. Following the progress of high-throughput DNA and RNA sequencing, novel tools are being developed to identify gene fusions [44,45,46,47,48,49]. While some of these methods showed high accuracy and speed, the sensitivity remains to be improved especially for low expressed transcripts [45]. In order to provide more insights in identification of tumor-specific neoantigens derived from gene fusions, Rathe et al. compared the fusion sequences provided by the deFuse algorithm [50] to the transcriptome generated by the Trinity method [51], and used NetMHCpan 4.0 [10] to predict neoepitope binders [52] from osteosarcoma patient samples. The authors were able to identify candidate neoantigens associated with fusions and found the frequency of fusion events to correlate with patient outcome. Although promising, additional studies are now required to experimentally validate the approach.

Early exploratory clinical trials uncovered the actionable potential fusion-neo-antigens [53,54,55]. In one trial, three patients with Philadelphia chromosome positive acute lymphoblastic leukemia were treated with BCR/ABL-specific CD8 T cells expanded from the patients’ peripheral blood mononuclear cells or from donors’ hematopoietic stem cells. Interestingly, BCR/ABL-specific T cells were increased in the bone marrow of all three patients post infusion, and all patients achieved a molecular or hematologic complete remission [56]. In solid tumors, targeting of the EWS/FLI-1 and PAX3/FKHR breakpoint regions in Ewing sarcoma and alveolar rhabdomyosarcoma patients through vaccination showed mixed results. In a first pilot study, the vaccinated patients did not show clinical benefit, nor did they develop a T cell response [57]. However, in a subsequent study, vaccinated patients had an increased overall survival and 25% developed vaccine-induced T cell responses [58]. The different outcomes between the two studies could be due to the different vaccine platforms used or the patient population. Indeed, the patients in the second study were in remission compared to an advanced cancer stage for the first study (Table 2). This may suggest that treating healthier patients with low/no tumor burden with vaccines may be more effective. 

Although these early results are encouraging, some challenges remain to be overcome in order to include gene fusion-derived neoantigens in clinical neoantigen-based immunotherapy pipelines. The sensitivity of fusion products identification would benefit from wide access to deep sequencing technologies. In addition, expanding validation sets from presented gene fusion-derived peptides to train neoantigen prediction algorithms would help prioritizing neoantigens from alternative sources (Table 1 and Table 2). 

## 3. RNA Aberrations

### 3.1. Alternative Splicing

Splicing is the essential step that creates mature mRNAs by removing introns from pre-mRNAs made of both introns and exons, and allows protein synthesis. Alternative splicing is a multiplexing process enabling the generation of multiple proteins from a single gene (different combination of exons), allowing protein diversity (Figure 1). Splicing events are involved in all major cell functions and, therefore, the splicing machinery or spliceosome is highly regulated.

Alternative splicing events (ASE) are detected at the transcriptome level and include skipped exons, alternative 5′ splice sites (donor), alternative 3′ splice sites (acceptor), retained intron, and mutually exclusive exon usage [59,60,61] (Figure 1). Computational methods based on RNA sequencing are used to detect and quantify ASE (reviewed elsewhere [62,63,64,65,66,67,68]). However, precise quantification of transcript isoforms remains challenging due to limited read length. The depth and quality of sequencing is also at play to improve the detection of novel transcripts and limiting false negative events.

Dysregulation of splicing events is involved in oncogenesis [61] and associated with drug resistance. Furthermore, tumor-specific ASEs have been used as predictive bio-markers for therapeutic response and/or clinical outcome in various cancers [69,70,71,72,73,74,75]. Mutations occurring in the spliceosome machinery, in addition to directly providing a source of SNV-neoantigens, also produce potential abnormal splicing events that may generate novel antigens. Kahles et al. identified MHCI epitopes derived from ASEs in breast and ovarian cancers from the TGCA immunopeptidomic database [76]. In these two cancer indications with relatively low SNV numbers, epitopes derived from ASEs were more abundant than from SNVs. In addition, ASE-derived peptides have been shown to be immunogenic in vitro [77], and in vivo with specific T cells found in the blood of cancer patients [78]. Some tumors also have an increased number of splicing events compared to normal tissue [76], suggesting that ASEs may generate neoantigens that are tumor-specific. Indeed, mutation of the splicing factor SF3B1 has been shown to induce tumor-specific ASE-derived neoepitopes that are recognized by CD8 T cells from uveal melanoma patients [79]. Altogether, these studies highlight that ASE-derived peptides can be immunogenic and tumor-specific, thus being suitable for neoantigen-based immunotherapy (Table 1 and Table 2).

Currently, most of the therapeutic approaches rely on pharmacological compounds targeting the spliceosome machinery. While targeting ASE-derived neoantigens for immunotherapy is attractive, several questions need to be addressed before their use in the clinic: (1) Can these novel epitopes generate strong and lasting anti-tumor responses? ASEs seem less expressed than SNVs [76], thus possibly limiting ASE-derived neoepitope presentation. In addition, some splicing events induce only minor changes to the protein sequence and may not bypass immune tolerance. Similarly to SNVs, evaluating the dissimilarity to self-peptides may improve prediction and selection of immunogenic ASE-neoantigens for targeted immunotherapies [35]. (2) How truly tumor-specific are the predicted ASE and are they not occurring in normal cells in a tissue-specific manner? (3) How many of ASEs are shared between tumor cells and/or patients? ASEs seem to mostly derive from passenger- rather than driver- mutations [76], suggesting that they are likely private (Table 1). 

### 3.2. Non-Coding Genomic Regions

Most of the human genome is considered as non-protein coding genes. With the development of sequencing technologies (WGS, RNA-seq, ChIP-seq, ATAC-seq, Hi-C, …), annotations for the non-coding genome have widely increased in the last decades. This led to discovery and characterization of RNA transcripts such as short and long non-coding RNAs (lncRNAs) or circular RNAs (Figure 1), and their role in regulating transcription, splicing, and translation [80]. Surprisingly, peptides derived from annotated non-coding sequences have been identified. In some instances, mislabeled annotations as “non-coding” seem to explain this observation [81,82]. Some of these regions have also been suggested to produce non-stable and thus non-functional proteins, leading to a quick degradation [83]. Other regions, known as pseudogenes, have recovered a lost protein-coding function in cancer cells [84], suggesting tumor-specificity to these “dark matter” antigens.

The recent advances in peptidomics and proteogenomics have increased the sensitivity of the techniques, thus detecting lower amounts of presented peptides [85]. However, most of these techniques refer to annotated proteins. It is only recently that new tools have emerged to identify unconventional antigens [86]. Interestingly, ribosome profiling has identified long non-coding RNA with both 5′ cap and polyA tails bound to ribosomes. Although the resulting translated proteins may not be functional, epitopes have been shown to be presented on the tumor cell surface [87]. Ribo-seq tool has also allowed identification of HLA-I presented peptides derived from small or novel unannotated open reading frames (smORF or nuORF) [83,88,89]. Importantly, these dark matter antigens have shown tumor-specificity to some extent [89,90] (Table 1). Using a new workflow combining immunopeptidomics, RNA-seq and Ribo-seq, Chong et al. suggested that 23% of the identified non-canonical HLA-Ip can be considered as tumor-specific [87]. These results, with other studies demonstrating tumor-specificity of HLA epitopes derived from non-coding regions [91] are encouraging for cancer patient immunotherapy. Another recent study, in a preclinical colorectal tumor model, showed delayed CT26 tumor growth in mice prophylactically vaccinated with peptides derived from the cryptic or “non-coding” transcriptome identified by mass spectrometry [92]. Although this study lacks definitive proof of specific T cell response, it suggests that these cryptic peptides can generate an anti-tumor response. Importantly, only a very limited number of these cryptic antigens showed anti-tumor effects, and the combination of three cryptic peptides were required for reducing tumor growth post vaccination, suggesting either limited presentation and/or weak T cell responses for each of the antigens. Similarly, only one of the identified non-canonical peptides from the Chong et al. study was found to be immunogenic [87]. Thus, sufficient clonality and quantity of these antigens from non-coding regions to generate efficacious anti-tumor T cell responses remains to be demonstrated before being clinically tested for immunotherapy in cancer patients (Table 2). Another concern is that non-functional proteins may be more easily subject to immunoediting, thus leading to tumor escape.

### 3.3. Transposable Elements

Transposable elements (TEs) or jumping genes are DNA-repetitive sequences integrated into the human genome that perform essential functions in driving genome evolution [93]. There are two different classes of TEs: (1) DNA transposons that mobilize through a DNA intermediate in a “cut and paste” mechanism, and (2) retrotransposons that undergo reverse transcription with a “copy and paste” mechanism. Although DNA transposons are part of the human genome, they are no longer active [94]. Retrotransposons are subdivided into two types: long terminal repeat (LTR) or non-LTR retrotransposons. LTR retrotransposons include human endogenous retroviruses (hERVs) derived in part from ancient retroviruses that infected germ cell progenitors, and the mammalian apparent LTRs retrotransposons (MaLRs). Non-LTR transposons include long interspersed nuclear elements (LINEs) that are autonomously active retrotransposons, or short interspersed nuclear elements (SINEs) and SINE-VNTR-Alu (SVAs), with both needing a specific protein for activation [95,96]. While most retrotransposons are silenced through DNA methylation, histone modifications, or RNA-mediated silencing, dysregulation can occur and lead to cancer initiation or progression through various mechanisms (reviewed elsewhere [96,97]). 

Genomic TE insertions are identified through whole-genome sequencing, RNA-seq or Ribo-seq, and specific bioinformatic tools showing variable sensitivity and specificity [98]. Most methods are designed for short-read sequencing, which limits the detection of TE’s repetitive sequences. The combination of both short-read and long-read sequencing data seem to increase the detection of both germline and somatic TEs [99]. However, the development of comprehensive pipelines combining different tools is recommended for higher performance [98,100] (Table 1).

Several studies have shown that the amount of TE transcripts and their derived peptides presented by HLA-I is abnormally increased in human cancer cells [101,102], and associated with tumor-infiltrating T cells as well as enhanced responses to ICB in cancer patients [102,103]. Immunogenicity of TE-derived epitopes was reported a long time ago in preclinical models and showed to protect against tumor challenge [104,105]. In humans, recognition of HERV antigens by CD8 T cells from healthy donor PBMCs has been demonstrated [106,107], as well as from cancer patients [108,109]. In addition, selective expression of HERV-E antigens has been associated with tumor regression in metastatic renal cell carcinoma [110]. These results suggest that TE-derived peptides are suitable for immunotherapy. Moreover, this targeted strategy may be used across patients since presented peptides can be found from conserved TE families (HERV, LINE, SINE, and SINE-VNTR-Alu) [101], and specific T cells against the same HERV antigens found in tumors from multiple patients [102] (Table 2). However, since TE can be expressed in normal cells [103] and many share sequence homologies, their immunogenicity may be limited. In addition, the expression pattern of TEs varies between cancer types [103] and since most computational tools seem to identify TE subfamilies with specificity [98], further development of computational workflows is required for high throughput TE identification and selection pipelines (Table 1). Interestingly, increased expression of TE-transcripts and TE-derived epitopes have been shown in cancer cells after in vitro treatment with DNA methylation inhibitors [101,111,112,113], suggesting that DNA-demethylating therapy (hypomethylating drugs such as azacytidine and decitabine) could be used in combination with immunotherapies. Further studies have shown synergistic anti-tumor effects with ICB therapies in preclinical models [114]. Epigenetic repression of TE-transcripts has also recently been associated with resistance to anti-PD1 therapy [115], and blocking the KDM5B–SETDB1 interaction could lead to effective anti-tumor responses mediated by TE-specific T cells [115,116]. Many epigenetic therapies are currently tested in clinical trials with some already approved for hematological malignancies [117], and it will be interesting to determine whether the objective clinical responses are associated with modulation of the immune response against TEs. 

## 4. Post-Translational Modifications

Tumor antigens can derive from post-translational modifications (PTM) that induce peptides that differ from the parental protein sequence. Different PTM have been involved as potential sources of candidate neoantigens such as glycosylation [118], phosphorylation [119,120,121,122], citrullination [123], or peptide splicing (Figure 1).

Glycosylation is the covalent attachment of a carbohydrate or glycan to a protein by a glycosyltransferase, and the most common PTM occurring in cells. Tumor cells present altered glycosylation patterns [124,125], and N- (Asn-linked) or O- (Ser/Thr-linked) glycosylations have been shown to generate neo- or overexpressed glyco-antigens that can be presented on the cell surface in many cancer types (reviewed in [126,127]) (Table 1). Altered glycosylation (and expression) of mucin 1 (MUC1) is one of the most studied PTM events and has been associated with several cancers [128]. Many therapeutic strategies targeting MUC1-derived truncated O-glycans such as Tn, sialyl-Tn, or Thomsen-Friedenreich (TF) antigens have been tested in clinical trials (phases 1 to 3), including dendritic cell, peptide-, or virus-based vaccines [128]. In contrast to early trials showing encouraging results, the most advanced trials showed mitigated results. No significant difference in overall survival was observed with non-small cell lung cancer (NSCLC) patients receiving Tecemotide (L-BLP25) peptide vaccine in a phase 3 trial [129]. In contrast, progression-free survival was significantly improved for patients receiving a modified vaccinia Ankara (MVA) expressing MUC1 and interleukin-2 (TG4010) plus chemotherapy compared to the control arm in a phase 2b/3 trial for advanced NSCLC. However, the survival benefit was only 0.8 months [130] (Table 2).

Cancer cells present dysregulated signaling pathways inducing an increased protein phosphorylation level and phosphopeptide presentation at the cell surface in various tumors [119,122,131,132]. Interestingly, phosphopeptides can be recognized by CD8 T cells [119,121] in a specific manner (in comparison to the non-phosphorylated counterpart) [119], and CD4 T cells [120] (Table 1). In a recent phase 1 clinical trial, 6 out of 15 melanoma patients showed evidence of CD8 T cell responses post vaccination with 2 phosphopeptides and adjuvants, including 2 pre-existing responses [133] (Table 2). While these results are encouraging, further studies are needed to demonstrate that targeting phospho-neoantigens for immunotherapy can generate anti-tumor activity in cancer patients. 

Citrullination is the conversion of arginine into citrulline by peptidylarginine deiminases, which can alter the protein structure. Thus, the interest for this PTM has recently increased and its evaluation as an immunotherapy target is ongoing. Citrullinated vimentin and enolase peptides have been shown to be presented on MHCII and recognized by CD4 T cells [134,135] (Table 1). Brentville et al. demonstrated that transduced tumor cells can present a citrullinated vimentin peptide on HLA-DR4 molecules, and that differential recognition by CD4 T cells occurs compared to the WT counterpart peptide [136]. Citrullinated vimentin peptide vaccines delayed tumor growth and increased survival rates of HLA-DR4 transgenic mice implanted with B16F1 tumors expressing HLA-DR4 [136] (Table 2). Interestingly, CD4 Th1 T cell responses from PBMCs from ovarian cancer patients and healthy donors were observed against citrullinated vimentin and enolase peptides [137]. While these results suggest promising candidates for immunotherapy, further studies are required to demonstrate tumor-specificity. In addition, the presentation of citrullinated peptides presented on MHCI remains to be demonstrated.

Finally, studies have identified CD8 T cells recognizing spliced peptides in renal cell carcinoma [138], in melanoma [139], or from EBV-B cells [140], thus suggesting their immunogenic potential (Table 2). Interestingly, Liepe et al. suggested that spliced peptides represent about 25% of the HLA-I peptidome of human cancer cell lines, thus increasing the possibility of novel source of antigens [141]. However, another study only estimated it to be 2–6% of the HLA-I ligandome [142], suggesting that further work is required for accurate identification of these events. While new identification tools and workflows have emerged in the past years [141,143,144,145], biological validations remain to be demonstrated (Table 1). In addition, the mechanism behind peptide splicing remains unclear. Some studies have suggested that the proteasome can form spliced peptides by ligation of two fragments from the same source protein sequence [138,139], with deamidation thus changing amino acid residues from asparagine to aspartate [146] or by transpeptidation and trimming that led to a spliced peptide with short C-terminal fragment [147]. However, very few spliced peptides have been validated so far, leading to the hypothesis that splicing peptide events are rare, and thus possibly not relevant for immunotherapy.

In conclusion, the shared nature of PTM neoantigens makes them interesting targets but many challenges remain to be addressed to prove the relevance for immunotherapy. Increasing the precision and sensitivity in identification of most of these events is required in order to accurately evaluate the frequency of PTM-derived presented peptides on MHC molecules. In addition, most PTM are commonly used in cell metabolism, and determining their tumor-specificity is essential. Nevertheless, as many of the identified PTM-neoantigen candidates were shared between tumors and patients [118,121,128,133,136], it seems important to pursue the efforts in characterizing tumor-specific PTM-derived peptides, as well as the development of predictive methods.

## 5. Conclusions

Early clinical studies using [SNV/small indels]-neoantigen based-vaccines have shown that T cell responses can be induced in patients but their clinical benefit is rather disappointing [2,148,149,150,151,152]. It is noticeable that the magnitude and breadth of the T cell responses induced by these vaccines is generally low, at least in the blood, and appears insufficient for efficacy. Improved neoantigen prediction algorithms as well as the design or superior vaccine platforms may help overcome this limitation. Alternatively, the quality of the T cell response may be suboptimal. Novel and improved methods to explore the contribution of alternative sources of neoantigens to further refine our understanding of the tumor antigen landscape, as well as the characterization of tumor-specific T cell responses in cancer patients developed spontaneously or after CIB treatment, will be critical to define the determinants of effective T cell responses beyond immunogenicity, and design improved immunotherapies.

Clonal neoantigens are expressed in a higher number of tumor cells, and the fraction of clonal neoantigens correlates with ICB response [153]. As driver mutations are more clonal than passenger mutations, as well as more likely to be shared between patients, they seem to be an ideal target. However, studies have shown that shared predicted neo-epitopes between patients are rare [154,155] and less presented by MHC molecules [155,156,157]. Nevertheless, clonal neoantigens seem to generate more CD8 T cell responses compared to subclonal neoantigens [158,159]. This may be of importance when choosing the source of neoantigens for immunotherapy as many RNA or PTM aberrations are not likely to be shared within the whole tumor, unless it is induced by an oncogenic event. 

The expression of neoantigens is a key immunogenic criterion. As DNA aberrations may lead to non-transcript events, most of the neoantigen prediction pipelines include filters for RNA expression levels. However, high throughput identification of translated products is challenging for all sources of neoantigens. Since presentation on MHCI by tumor cells is required for CD8-induced tumor cell killing, several clinical neoantigen selection pipelines include direct neoantigen identification through mass spectrometry. Nevertheless, identification of some neoantigens by MS such as “non-coding”, TE- or PTM-derived peptides remains to be improved before being included in clinical pipelines. In addition, lack of tumor recognition by SNV-neoantigen-specific CD8 T cells has been linked to the insufficient amount of presentation [160]. Thus, development of new quantitative approaches such as the TOMAHAQ-targeted MS [9] are needed to correlate the amount of presented neoantigens and CD8 T cell-induced anti-tumor efficacy, especially for comparing sources of neoantigens. 

Another important consideration is the likelihood of neoantigen expression loss that may differ between sources. Indeed, HLA loss is observed in cancers and suggested to lead to tumor immune evasion [161]. Thus, neoantigens binding to multiple HLA may offer a therapeutic advantage. In addition, potent and dominant neoantigen-specific T cell response may favor the loss of neoantigen expression due to immune pressure [162]. Therefore, it remains to be shown that immunotherapy targeting neoantigens derived from more disruptive aberrations would generate long and lasting memory T cell responses.

An additional aspect modulating neoantigen quality is the platform used for immunotherapy. Many platforms have demonstrated potent vaccine-induced neoantigen-specific T cell responses in clinical trials, including RNA [149,152], dendritic cell [148,163,164,165], or peptide [150]. However, specific T cell responses can vary with neoantigen-based therapies [35,166], and some sources of neoantigen may have limited platform options. For example, some PTM-derived neoantigens would preferably require peptide-based vaccines or adoptive cell therapy, which have shown manufacturing challenges and production delays. 

Finally, studies suggest that T helper cells are required to generate an efficacious anti-tumor-specific CD8 response. In two different preclinical tumor models (T3 MCA-induced sarcoma and SMA560 glioma), anti-tumor activity of SNV-neoantigen specific CD8 T cells could only be observed with MHCII neoantigen co-expression by tumor cells [167,168]. In addition, Swartz’s study suggests that vaccines encoding a non-tumor-specific MHCII-restricted antigen associated with MHCI neoantigen may be sufficient to generate anti-tumor responses [168], which would facilitate vaccine design and manufacturing. While additional studies need to confirm these findings and the mechanisms, the type (MHCI and/or MHCII) of T cell response generated by different sources of neoantigen may not be equal. It is important to note that combined with the vaccine platform, which also impacts immune cells differently, the balance between CD4 and CD8 T cell responses may be highly modified.

**Table 1:** Summary of neoantigen reactivities.

Alterations	Presentation	Immunogenicity	Shared between Patients	Tumor-Specificity	Tumor Alteration Burden	Main Challenges
SNV/indels	MHCI, MHCII	CD8, CD4	Mostly private	Yes	Low to high depending on cancer type	Immunogenicity (similarity to self)
Gene fusion	MHCI, MHCII [53,54,55]	CD8, CD4 [53,54,55,56]	Yes	Yes	Low	Identification, prediction
Alternative splicing	MHCI, MHCII [76,169]	CD8, CD4 [77,78,79,169]	TBD	TBD	TBD	Identification, tumor-specificity
Non-coding genomic regions	MHCI, MHCII [83,86,87,88,89]	CD8 [87]	Yes	Yes	TBD	Immunogenicity, tumor-specificity
Transposable Elements	MHCI, MHCII [104,105,106,107,108,109]	CD8, CD4 [104,105,106,107,108,109]	Yes	No	TBD	Identification, tumor-specificity
Glycosylation	MHCI [118]	CD8 [118]	Yes	TBD	Low	Identification, prediction, tumor-specificity
Phosphorylation	MHCI, MHCII [119,120,121,122]	CD8, CD4 [119,120,121,122]	Yes	TBD	Low	Identification, prediction, tumor-specificity
Citrullination	MHCII [134,135,136]	CD4 [134,135,136]	Yes	TBD	Low	Identification, prediction, tumor-specificity
Peptide splicing	MHCI	CD8 [137,138,139]	TBD	TBD	TBD	Identification, prediction, tumor-specificity

SNV: Single nucleotide variant; TBD: To be demonstrated.

**Table 2:** Clinical development stage of neoantigen-targeted therapies.

Alterations	Altered Molecule	Identification	Prediction	Most Advanced Development Stage	Example
SNV/indels	DNA	WES + RNA-seq	Available (many)	Phase 1/1b; several ongoing Phases 2/3	Immunogenic responses observed in patients receiving peptide/DC/mRNA vaccines; or adoptive T cell therapy in different cancer types [147,148,149,170,171]
Gene fusion	DNA	WES + RNA-seq	Available (few)	Phase 2	Immunogenic response but no clinical efficacy observed in patients with CML following bcr-abl peptide vaccination [172]
Alternative splicing	RNA	RNA-seq, Ribo-seq	Available (few)	Preclinical	CD8 T cell recognition of the mutated splicing factor SF3B1 in patients with uveal melanoma [79]
Non-coding genomic regions	RNA	RNA-seq, Ribo-seq	NA	Preclinical	Delayed tumor growth of CT26 tumors following cryptic peptide vaccination without proof of specific T cell response [92]
Transposable Elements	RNA	WES + RNA-seq, Ribo-seq	Available (few)	Preclinical ongoing Phase 1	Recognition of HERV antigens by CD8 T cells from patients [108,109] HERV-E TCR Transduced Autologous T Cells in Metastatic Kidney cancer patients (*)
Glycosylation	Protein	Mass spectrometry	NA	Phase 3	No overall survival benefit with L-BLP25 peptide vaccine in NSCLC patients [129]; Improved progression free survival post TG4010 vaccine + chemotherapy in NSCLC patients [130]
Phosphorylation	Protein	Mass spectrometry	NA	Phase 1	Some specific CD8 T cell responses were observed in melanoma patients who received pIRS2 and pBCAR3 peptide vaccines [133]
Citrullination	Protein	Mass spectrometry	NA	Preclinical	Delayed B16F1 tumor growth in HLA-DR4 transgenic mice following citrullinated peptide vaccination. Citrullinated-specific CD4 T cell responses also observed in PBMC from ovarian cancer patients [136]
Peptide splicing	Protein	Mass spectrometry	NA	Preclinical	Spliced peptide identified and recognized by CD8 T cells in renal cell carcinoma [137] or melanoma [138] patients, and from EBV-B cells [139]

SNV: Single nucleotide variant; WES: Whole exome sequencing; seq: sequencing; NA: Not available. * ClinicalTrials.gov.
